# Dr. Answer AI for prostate cancer: Clinical outcome prediction model and service

**DOI:** 10.1371/journal.pone.0236553

**Published:** 2020-08-05

**Authors:** Mi Jung Rho, Jihwan Park, Hyong Woo Moon, Chanjung Lee, Sejin Nam, Dongbum Kim, Choung-Soo Kim, Seong Soo Jeon, Minyong Kang, Ji Youl Lee

**Affiliations:** 1 Catholic Cancer Research Institute, College of Medicine, The Catholic University of Korea, Seoul, Republic of Korea; 2 Department of Biomedicine & Health Sciences, College of Medicine, The Catholic University of Korea, Seoul, Republic of Korea; 3 Department of Urology, Seoul St. Mary’s Hospital, College of Medicine, The Catholic University of Korea, Seoul, Republic of Korea; 4 LifeSemantics, Seoul, Republic of Korea; 5 Department of Urology, Asan Medical Center, University of Ulsan College of Medicine, Seoul, Republic of Korea; 6 Department of Urology, Samsung Medical Center, Sungkyunkwan University School of Medicine, Seoul, Republic of Korea; 7 Department of Health Sciences and Technology, SAIHST, Sungkyunkwan University, Seoul, Republic of Korea; The Cancer Institute of New Jersey, Robert Wood Johnson Medical School, UNITED STATES

## Abstract

**Objectives:**

The importance of clinical outcome prediction models using artificial intelligence (AI) is being emphasized owing to the increasing necessity of developing a clinical decision support system (CDSS) employing AI. Therefore, in this study, we proposed a “Dr. Answer” AI software based on the clinical outcome prediction model for prostate cancer treated with radical prostatectomy.

**Methods:**

The Dr. Answer AI was developed based on a clinical outcome prediction model, with a user-friendly interface. We used 7,128 clinical data of prostate cancer treated with radical prostatectomy from three hospitals. An outcome prediction model was developed to calculate the probability of occurrence of 1) tumor, node, and metastasis (TNM) staging, 2) extracapsular extension, 3) seminal vesicle invasion, and 4) lymph node metastasis. Random forest and k-nearest neighbors algorithms were used, and the proposed system was compared with previous algorithms.

**Results:**

Random forest exhibited good performance for TNM staging (recall value: 76.98%), while k-nearest neighbors exhibited good performance for extracapsular extension, seminal vesicle invasion, and lymph node metastasis (80.24%, 98.67%, and 95.45%, respectively). The Dr. Answer AI software consisted of three primary service structures: 1) patient information, 2) clinical outcome prediction, and outcomes according to the National Comprehensive Cancer Network guideline.

**Conclusion:**

The proposed clinical outcome prediction model could function as an effective CDSS, supporting the decisions of the physicians, while enabling the patients to understand their treatment outcomes. The Dr. Answer AI software for prostate cancer helps the doctors to explain the treatment outcomes to the patients, allowing the patients to be more confident about their treatment plans.

## Introduction

Currently, it has become easier to collect and use electronic medical record (EMR) data from multiple hospitals because of the increased availability of multi-center clinical data provided by hospitals. In addition, owing to the growing necessity for developing a clinical decision support system (CDSS) that employs artificial intelligence (AI), the importance of predictive models using AI has been emphasized. Therefore, several researchers have attempted to develop predictive models using AI in their studies on prostate cancer (PCa) [1–3].

In South Korea, a large-scale government-supported project employing large-scale multi-organization data has also been initiated in 2018 by the National IT Industry Promotion Agency (NIPA) [4]. The “Korea Data and Software-driven Hospitals (K-Dash)” is a collaborative consortium of 26 hospitals and 22 companies that are developing “Dr. Answer”, which is an AI solution for eight diseases such as cardio-cerebral vascular disease, heart disease, breast cancer, colon cancer, PCa, dementia, epilepsy, and childhood incurable genetic diseases. The “PROstate Medical Intelligence System Enterprise-Clinical, Imaging, and Pathology (PROMISE CLIP)” is one of the NIPA-supported projects for PCa undertaken by K-Dash. This project has been addressing the medical requirements of PCa since April 1, 2018.

PCa is difficult to diagnose and requires complicated treatments depending on the condition of a patient. Additionally, it is difficult to provide detailed explanations of the prognosis to the patients. Thus, an AI-based technique for predicting the outcome of PCa is suitable as a CDSS for both physicians as well as PCa patients. The PROMISE CLIP project has developed four prediction models as follows: (1) Prediction of treatment outcomes after a definitive surgery, which helps to define an ideal patient population for aggressive follow-up or early postoperative ancillary treatment. (2) Prediction of pathologic outcomes to help patients and clinicians to choose an optimal treatment option. (3) Accurate interpretation of multiparametric magnetic resonance imaging (MRI). (4) Precise digital pathology of PCa to improve accuracy, reduce human error, and increase reproducibility [5].

In this study, we attempted to develop an outcome prediction model using the PCa clinical data of patients treated with radical prostatectomy. In addition, we developed a CDSS according to the clinical outcome prediction model for PCa.

## Methods

### Target user

The Dr. Answer AI software (SW) for prostate cancer is targeted towards patients diagnosed with PCa before undergoing a radical prostatectomy. The patients could be provided with a tumor, node, and metastasis (TMN) staging service and be informed of the PCa-related risk factors before the radical prostatectomy. Thereafter, they could determine the direction of treatment and also predict the outcome of the radical prostatectomy.

### Clinical outcome variables of clinical outcome prediction model

In this study, the clinical outcomes indicated the probability of the occurrence of 1) TNM classification, 2) extracapsular extension (ECE), 3) SVI, and 4) lymph node metastasis. They were determined with the assistance of three urologists based on the importance of clinical outcomes.

The PCa is staged using the TNM classification of cancer staging. It separately assesses the tumor, lymph nodes, and secondary cancer (metastases: M). In this study, we used the TNM classification as the first prediction outcome.

ECE refers to the growth or spread of tumor cells outside the lymph node capsule [6]. Models predicting extracapsular extension in PCa that are based on nomograms have been proposed earlier [7,8]. The occurrence of ECE in regional lymph nodes is one of the high-risk adverse pathological features in patients suffering from oropharyngeal squamous cell carcinoma [8]. In this study, ECE was used as the second prediction outcome.

SVI refers to the presence of PCa in the areolar connective tissue, which are present around the SVs and outside the prostate [9]. Previous studies have provided definitions of SVI: 1) PCa penetrating the muscular wall of the SV, 2) PCa must involve the extraprostatic portion of the SV, 3) cancer in the prostate and SV is usually directly contiguous and 4) a minority of tumors have noncontiguous metastases to the SV [10]. We have used SVI as the third prediction outcome.

Finally, metastatic lymph nodes are those lymph nodes that contain cancer cells, which can enable the cancer to spread from the primary tumor to other locations. It has been reported that the presence of lymph node metastases is an important independent prognostic factor for predicting deaths due to cancer. Moreover, previous studies have discovered that the lymph node metastases are associated with tumor recurrence and cancer-specific mortality [11]. A majority of cancer-specific deaths occur as a result of metastasis [12]. Thus, the metastatic lymph nodes are important in our prediction model.

### Algorithm based on random forest and k-nearest neighbors

In this work, algorithms, such as random forest (RF) and k-nearest neighbors (KNN), and logistic regression were applied to develop the Dr. Answer AI SW for PCa. Weka 3.8.3 and Python 3.7 were used as the machine learning programs. 12 clinical variables were used as input data (as listed in Table 1), including the age at diagnosis, family history, and Gleason score.

**Table 1:** 12 input variables.

No	Variable	Range	
1	Age at diagnosis	1: under 40	7: 65–69
		2: 40–44	8: 70–74
		3: 45–49	9: 75–79
		4: 50–54	10: 80–84
		5: 55–59	11: Over 85
		6: 60–64	
2	BMI	Weight / Height (cm: 130~200)* Height (kg: 30~200)	
3	Marital status	1: Single	4: Bereavement
		2: Marriage	99: etc.
		3: Divorce	
4	Education	1: Uneducated	5: University graduate
		2: Elementary school graduate	6: Graduate degree
		3: Middle school graduate	7: etc.
		4: High school graduate	
5	Smoking	1: Nonsmoker	3: Smoker
		2: Ex-smoker	99: etc.
6	Alcohol	1: Abstain from alcohol	99: etc.
		2: Drunker	
7	Family history of PCa	0 None	2: Family history with second cousin
		1: Family history with first cousin	99: etc.
8	Initial PSA value		
9	Gleason Grade Group	1: 3 + 3 = 6	4: 4 + 4 = 8
		2: 3 + 4 = 7	5: Gleason sum ≥ 9
		3: 4 + 3 = 7	
10	Maximum positive core(%)	0–100 (%)	
11	HGPIN(High-grade prostatic intraepithelial neoplasia)	0: No	1: Yes
12	Core ratio	Core ratio = Number of positive cores / Total cores	

Since its introduction in 2001, RF has been a popular technique that involves the aggregation of several decision trees, and has provided a reduction in the variance as compared to that using single decision trees [13]. The overall prediction obtained using RF is an average of the predictions from individual trees. Moreover, RF predicts the test data using an unweighted average over the collection by aggregating the built trees. RF has generalizability and robustness in overall performance and does not result in overfitting problems [14].

Meanwhile, KNN is a simple algorithm, first proposed in the early 1970s, that stores all the cases and classifies new cases based on a similarity measure. It is an effective model that is widely employed for data mining [15], and it functions effectively as a nonparametric model for classification and regression [16]. It is also commonly used in research on PCa [15,17–19].

Here, the data were divided into two sets, i.e., the training dataset and the test dataset. The seven training datasets were matched to three test datasets, and the study achieved a prediction performance over 10-fold cross validation.

### Ethics

The study was performed in accordance with the Declaration of Helsinki and was approved by the Institutional Review Board of Catholic University (IRB number: KC18SNDI0512), Samsung Medical Center (IRB number: SMC201807069001), Bundang Seoul University Hospital (IRB number: B1808486102), and Asan Medical Center (IRB number: 2018–0963). We waived the requirement for informed patient consent from IRB board.

## Results

### Input variables for clinical outcome prediction model

To develop the clinical outcome prediction model, 12 input variables related to PCa were used; the variables were 1) age at diagnosis, 2) BMI, 3) marital status, 4) education, 5) smoking, 6) alcohol, 7) family history of PCa, 8) initial prostate specific antigen (PSA) value, 9) Gleason grade group, 10) maximum positive core, total cores, 11) high-grade prostatic intraepithelial neoplasia(HGPIN), 12) core ratio(Number of positive cores / Total cores).

We collected 7,128 cases of anonymized PCa patients after the radical prostatectomy treatment: 1,723 were from hospital C, 2,751 from hospital S, and 2,654 from hospital A (Table 2). These three university hospitals are located in Seoul, South Korea. The Gleason mean ranges from 6.89–7.13, and the initial PSA mean ranges from 7.79–14.14. Most of the PCa patients were in the age group of 60–70 years.

**Table 2:** Demographic results from multiple organizations (n = 7128).

Organization		A	S	C
**Initial PSA value (mean (SD))**		7.79 (9.73)	8.63 (10.01)	14.14 (47.58)
**Gleason sum (mean (SD))**		7.06 (0.91)	7.13 (0.86)	6.89 (0.9)
**Age at diagnosis(%)**	**<40**	3 (0.001)	1 (0.000)	2 (0.001)
	**40–44**	13 (0.005)	1 (0.000)	3 (0.002)
	**45–49**	30 (0.011)	6 (0.002)	18 (0.010)
	**50–54**	139 (0.052)	113 (0.041)	59 (0.034)
	**55–59**	344 (0.130)	368 (0.134)	181 (0.105)
	**60–64**	571 (0.215)	688 (0.250)	352 (0.204)
	**65–69**	706 (0.266)	764 (0.278)	500 (0.290)
	**70–74**	618 (0.233)	676 (0.246)	446 (0.259)
	**75–80**	222 (0.084)	134 (0.049)	156 (0.091)
	**80–84**	6 (0.003)	0 (0.000)	8 (0.003)
**Total**	**7,128**	**2,654**	**2,751**	**1,723**

*SD: Standard deviation.

There were missing values for each outcome variable; the number of missing values were: 1) TNM classification (T = 1,847, N = 1,898, M = 1,896), 2) ECE (n = 260), 3) SVI (n = 218), and 4) lymph node metastasis (n = 533) (Table 3). Finally, each algorithm was developed with the final cases that excluded the missing values for each variable, as follows: 1) TNM classification (T = 5,281, N = 5,230, M = 5,232), 2) ECE (n = 6,868), 3) SVI (n = 6,910), and 4) lymph node metastasis (n = 6,595).

**Table 3:** Final outcome variables for algorithm.

Output Variable		Total = 7,128	Final Cases
T (tumor)	T1	22	5,281
	T2	3,500	
	T3	1,713	
	T4	42	
	TX	4	
	Missing value	1,847	
N (node)	N0 = No	3,942	5,230
	N1 = Yes	153	
	NX = N0 after surgery	1,135	
	Missing value	1,898	
M (metastasis)	M0	5,224	5,232
	M1	8	
	Missing value	1,896	
Extracapsular extension	No	4,554	6,868
	Yes	2,314	
	Missing value	260	
Seminal vesicle invasion	-No	6,031	6,910
	-Yes	879	
	Missing value	218	
Lymph node metastasis	-No	4,856	6,595
	-Yes	1,739	
	Missing value	533	

The final analysis method was chosen by comparing each of the algorithms (Table 4). The RF exhibited a good performance in TNM staging (recall: 76.98%). Furthermore, KNN exhibited a good performance for ECE, SVI, and lymph node metastasis (80.24%, 98.67%, and 95.45%, respectively). RF and KNN also exhibited a good performance using the synthetic minority oversampling technique (SMOTE), over 10-fold cross-validation. The SMOTE is an over-sampling method and an algorithm used in the framework of learning from imbalanced data [20,21]. Since its introduction in 2002, it has proven to be successful in various domains. In this study, there were small samples of occurrence of 1) TNM classification, 2) ECE, 3) SVI, and 4) Lymph node metastasis, and thus, this data was imbalanced.

**Table 4:** Results of algorithm performance.

No	Algorithm	TNM classification (%)	ECE (%)	SVI (%)	Lymph node metastasis (%)
1	Random Forest (RF)	*76*.98*	68.17	88.62	91.51
2	Support vector machine	27.85	50.31	52.26	83.60
3	Ridge Regression	63.57	58.16	60.56	87.76
4	AdaBoost	67.69	65.01	82.75	89.56
5	Gaussian NB	6.13	69.78	81.08	87.05
6	Gradient Boosting	76.52	64.84	86.56	90.04
7	K-Nearest Neighbors (KNN)	72.64	***80*.*24***	***98*.*67***	***95*.*45***

*: Recall value.

### SW structure and function

**Fig 1:**
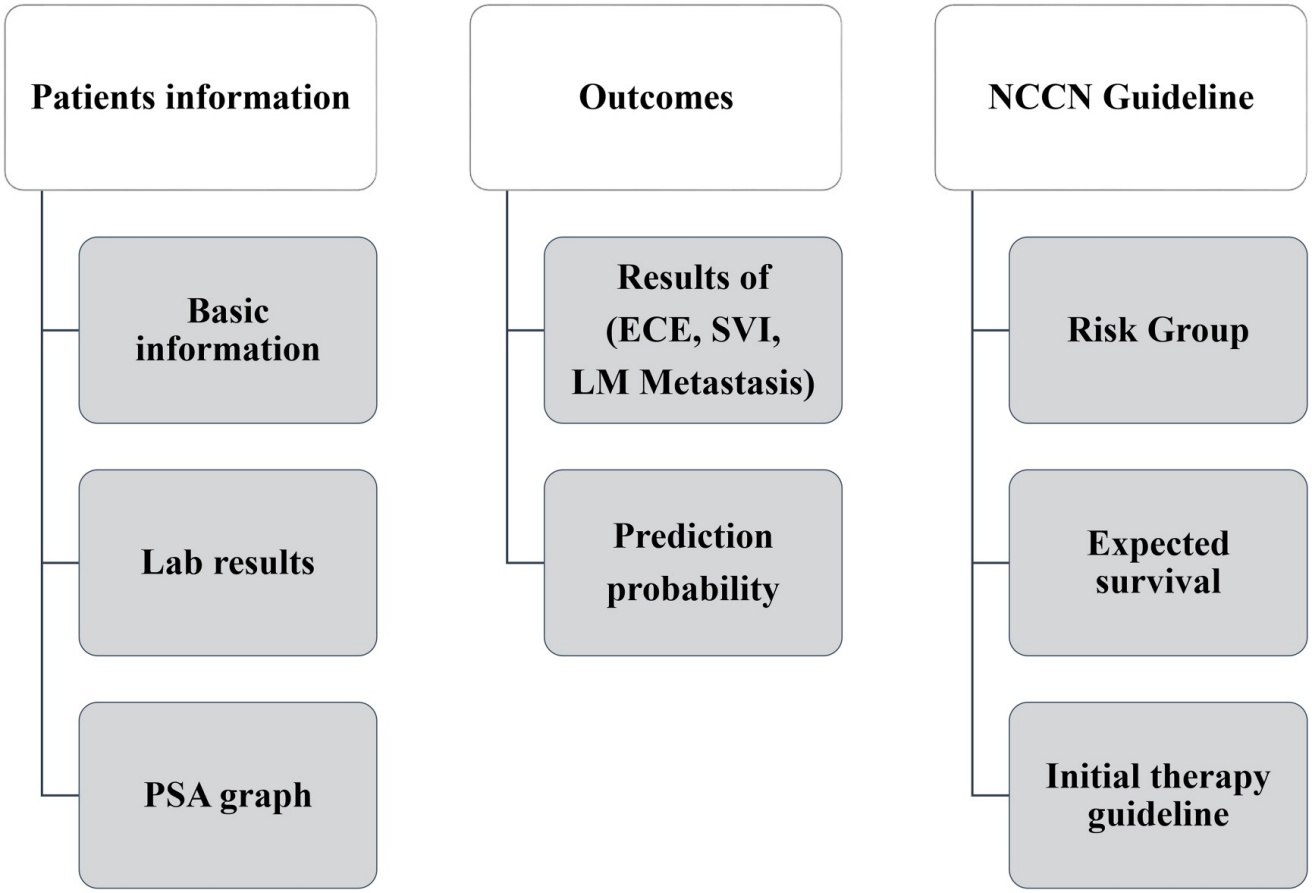
SW structure.

The Dr. Answer AI SW for PCa was developed according to our clinical outcome prediction model. It consists of three primary service structures: patient information, clinical outcome prediction, and outcome according to the National Comprehensive Cancer Network (NCCN) guidelines (Fig 1).

The patient information service structure could check the name, age at diagnosis, and family history of the patient. The clinical outcome prediction service structure could check clinical outcome results through computer tomography (CT), bone scan, and MRI images. There was a probability of occurrence in TNM staging, ECE, SVI, and lymph node metastasis.

The clinical outcomes were combined, and the patients were divided into risk groups according to the NCCN guidelines. This service provides an initial guideline for therapies.

### Service process

**Fig 2:**
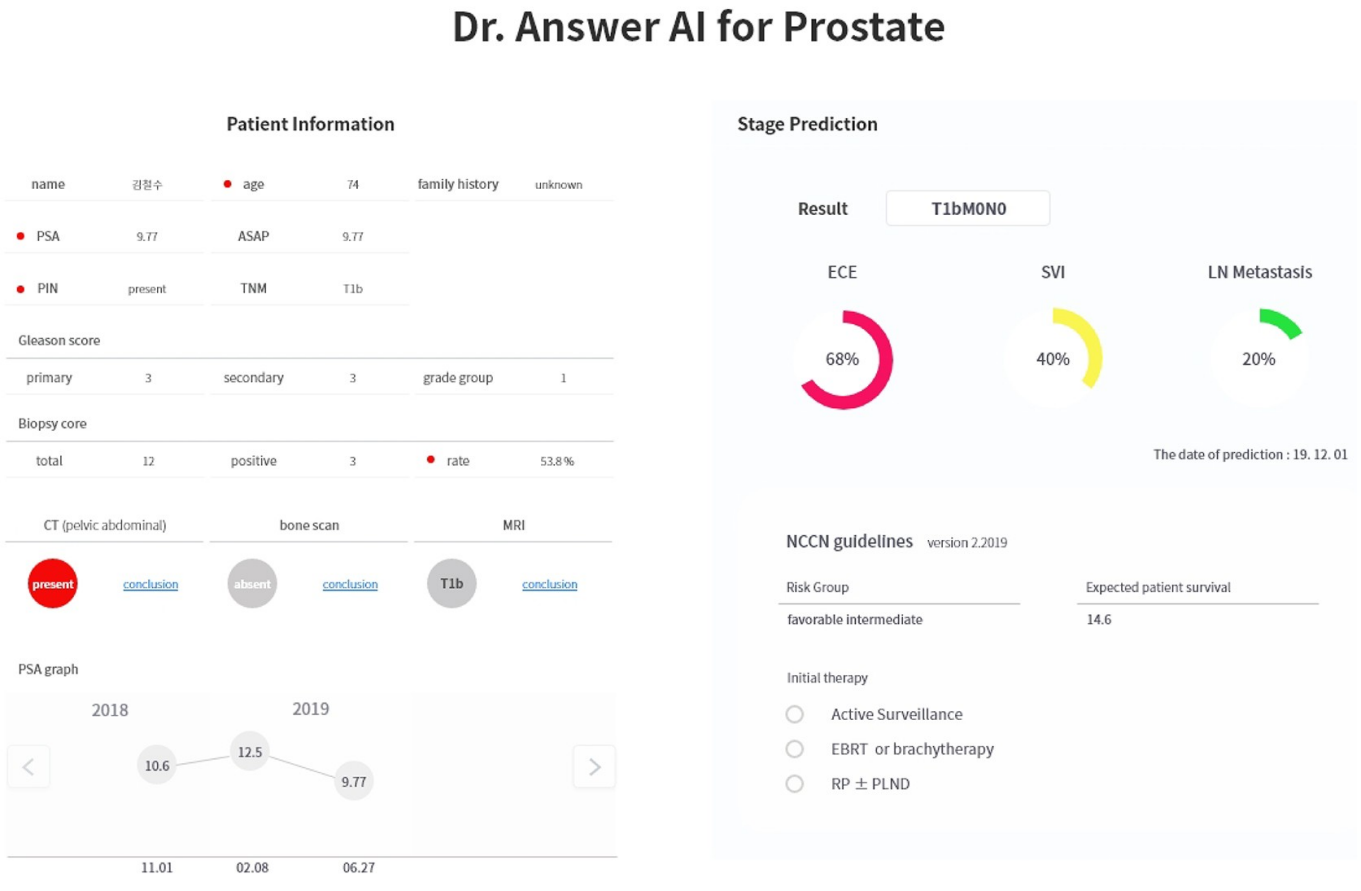
Main service screen. *red point: risk factor in service screen.

The service shown in section 1 of Fig 2 provides the basic patient information, consisting of four values: 1) age at the time of diagnosis, 2) BMI, 2) drinking status, and 4) smoking preference. The service shown in section 2 of the figure provides the EMR lab results, such as PSA, Atypical Small Acinar Proliferation(ASAP), PIN, TNM, Gleason score, biopsy score, CT, bone scan, and MRI images.

The service shown in the third section provides a trend graph of the lab results of PSA time series. Thereafter, this service provided the probability of occurrence of 1) TNM staging, 2) ECE, 3) SVI, and 4) lymph node metastasis according to our prediction algorithm. The probability of occurrence is provided using a color graph in section 4 of Fig 2. Finally, this service presented the risk group, expected survival of the patients, and initial therapy guidelines as per the NCCN guidelines.

**Fig 3:**
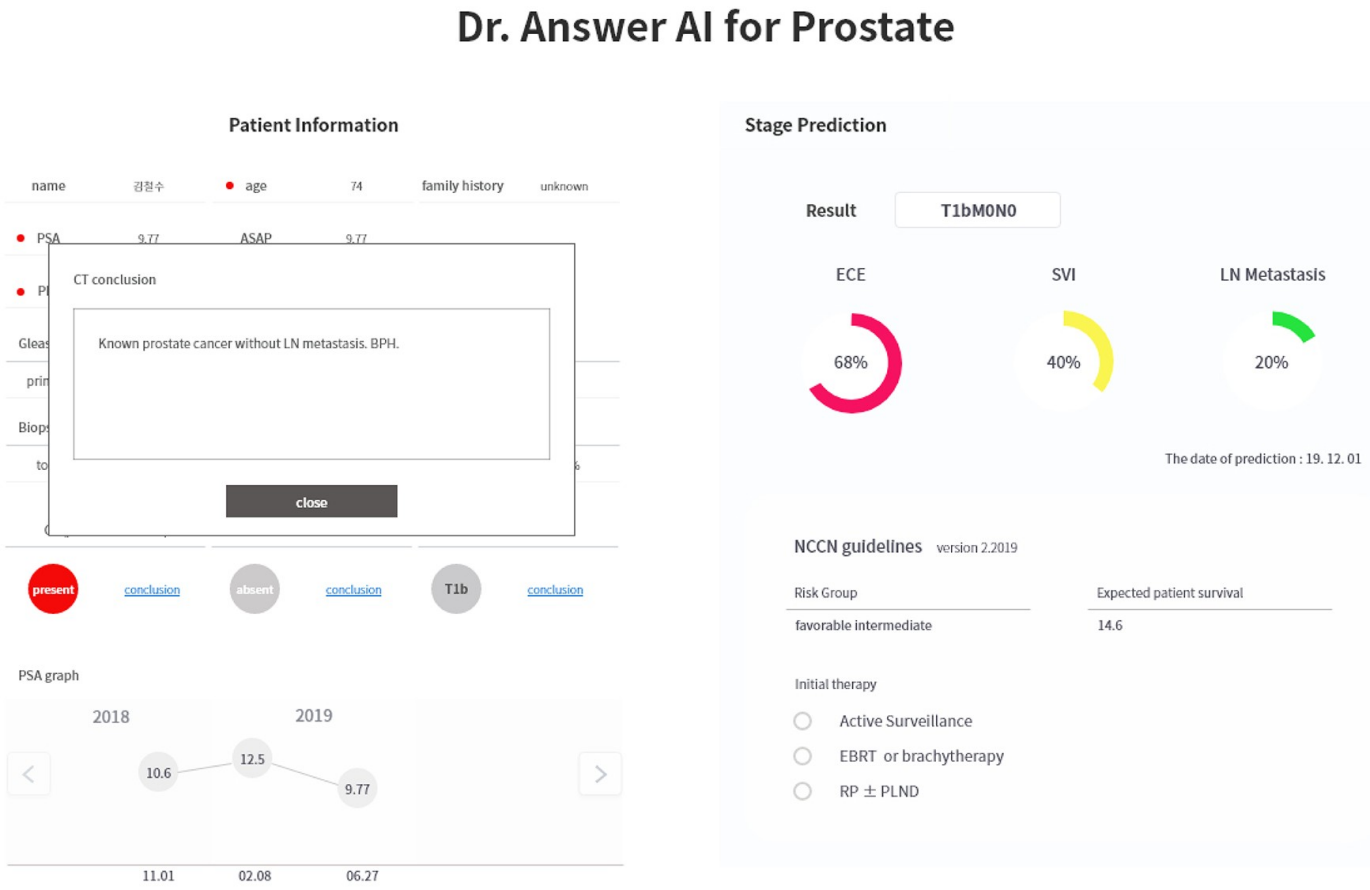
Prediction outcome screen.

The service received the patient data from the EMR and provided the results on a screen (Fig 3). In the case of CT, bone scan, and MRI images, the lab results could be found in detail using the conclusion button on the service page. The data recorded in this service were automatically recalled and saved during a product launch and shutdown. After using the service, the X button of the program was pressed to terminate the service.

### Patients’ experience

Our SW is targeted towards patients that have been diagnosed with PCa and have not undergone radical prostatectomy. Before radical prostatectomy, the patient can be provided with the TMN staging service and be informed of the risk factors related to PCa. This can help in determining the course of treatment and predict the outcome of the radical prostatectomy. The results of TMN staging and PCa related risk factors were expressed using a proportion. Understanding these results could be beneficial to PCa patients; the Dr. Answer AI SW for PCa provided results to the patients using a website and printed reports.

## Discussion

In this study, an outcome prediction model was developed using the multicenter clinical data of PCa with radical prostatectomy. The proposed SW, which is based on the clinical outcome prediction model for PCa treated with radical prostatectomy, is a part of the PROMISE CLIP-initiated Dr. Answer project [4], which began to address the medical demands of PCa. The proposed SW is targeted towards patients diagnosed with PCa, who have not undergone radical prostatectomy, as well as physicians. LifeSemantics Corp., which has an experience of developing a mHealth management platform for patients [22–24], has contributed to the development of the Dr. Answer AI SW for PCa.

PCa is difficult to diagnose and it requires complicated treatments depending on the condition of the patient. Moreover, it is difficult to explain the prognosis to the patients as the physicians, including the Korean physicians, may not have sufficient time to explain the treatment outcomes in detail. It has been reported that the shortage of the physicians’ time is a negative factor that causes overcrowding in outpatient healthcare [25]. According to the results of a previous survey, the average treatment time per patient in a Korean general hospital is 6.2 min. However, this should be increased to at least 8.9 min to increase patient satisfaction [26]. Therefore, there is a need for a CDSS that can provide effective treatment for PCa and support the short treatment time. The proposed SW could address these issues.

In addition, it has been reported that the patients are more satisfied when the physicians employ a patient-centered approach during consultations [27], which could be enabled using the proposed technique for predicting PCa outcomes. This could improve the patient satisfaction levels during consultations and treatments.

The clinical outcome prediction model could also function as a CDSS, supporting the decisions of doctors and helping the patients in understanding their treatment outcome. Furthermore, the Dr. Answer AI SW for PCa also enabled the doctors to clearly explain the treatment outcomes to patients; thus, allowing patients to have confidence in their treatment plans. The results of our service, indicating the TMN staging and PCa-related risk factors, have been provided using proportions and graphics; in addition, the patients can obtain a printed report directly from the physician as well as access the reports on the website of the Dr. Answer AI SW for PCa. Thus, the patients can check the PCa-related risk factors and treatment outcomes at any time, which puts them at ease during future treatments. Furthermore, the patients can determine the course of treatment and predict the outcome of radical prostatectomy. According to previous studies, the PCa decision aids have been reported to increase knowledge, reduce conflicts, and increase the active role of a patient during the decision-making process [28–30]. This is because the decision aids provide the user with detailed information and also help them to determine their preferred treatment [31]. While diverse CDSSs and PCa decision aids have been developed owing to the evolution of technology, the proposed clinical outcome prediction model could assist patients in the PCa treatment decision and compliance.

The proposed model is an integrated one based on AI, that employs the RF and KNN algorithms. In this study, we focused on the probability of occurrence of 1) TNM staging, 2) ECE, 3) SVI, and 4) lymph node metastasis. Each variable is important when predicting the PCa prognosis and treatments. According to a systematic review, ECE, SVI, and lymph node metastasis were statistically significant for BCR free survival [32]. Previous predictive models and related studies on each of the variables have not provided combined results [6,7,33–35]. In addition, previous research provided oncologic outcomes such as survival rate and biochemical recurrence. Although survival rate and biochemical recurrence are important outcomes, there are limitations in explaining the treatment outcomes to the patients in detail. We developed an integrated clinical prognosis prediction model that can predict various treatment outcomes such as 1) TNM staging, 2) ECE, 3) SVI, and 4) lymph node metastasis. The predictive rate of each variable is also high, which is considered to be useful clinically. Thus, the Dr. Answer AI SW for prostate cancer helps the doctors to explain the treatment outcomes to patients, instilling confidence in patients regarding their treatment plans.

However, there are certain limitations in this study. The SMOTE model was used because of the imbalanced data and the limited outcome of the sample data. Future studies should focus on developing the model using balanced data such that sizeable outcome data can be collected. We plan to test the utility of the proposed system via user questionnaires for patients and physicians, and update our SW based on the results. Finally, we focused on probability of occurrence of 1) TNM staging, 2) ECE, 3) SVI, and 4) lymph node metastasis as a clinical outcome. Future studies should focus on oncologic outcomes including survival rate and biochemical recurrence.

Despite these limitations, the clinical outcome prediction model could effectively act as a CDSS and support the decisions of the physicians as well as support the patients to understand their treatment outcomes. The Dr. Answer AI SW for PCa enables the doctors to explain the treatment outcome to the patients, thereby instilling confidence in patients regarding their treatment plans.

## Supporting information

S1 Data (PDF)
